# Supplementary material for: Methylation risk score of C-reactive protein associates sleep health with related health outcomes
Source: Commun Biol. 2025 May 28;8:821. doi: 10.1038/s42003-025-08226-1 (PMC12119824; doi:10.1038/s42003-025-08226-1)
Supplement: Supplementary file 1 — Supplementary information [file 42003_2025_8226_MOESM1_ESM.pdf]

Methylation risk score of C-reactive protein associates sleep health with related health outcomes

Supplementary information

Supplementary Methods: PRS Development 3
Supplementary Tables 5
Table S1. Demographic characteristics of MESA 5
Table S2. SNP counts in polygenic risk score (PRS) construction for MESA 5
Table S3. Association analysis for polygenic risk score (PRS)-CRP and blood CRP level in MESA 6
Table S4. Polygenic risk score (PRS) effect size obtained using MESA cohort 6
Table S5. SNP counts in ancestry-specific PRS construction in HCHS/SOL 7
Table S6. Background-specific effect estimates for MRS-CRP and PRS-CRP in HCHS/SOL 7
Table S7. Association analysis for obstructive sleep apnea (OSA) cases with and without excessive daytime sleepiness (EDS) in HCHS/SOL 8
Table S8. CpG sites selected by lasso penalized regression for respiratory event index 9
Table S9. CpG sites selected by Lasso penalized regression for minimum oxygen saturation 11
Table S10. CpG sites selected by Lasso penalized regression for diabetes 11
Table S11. CpG sites selected by Lasso penalized regression for Hypertension 12
Table S12. One sample Mendelian Randomization analysis for blood CRP and MRS-CRP as potential causes 13
Supplementary Figures 14
Figure S1. Density plots for original and log-transformed blood CRP levels in HCHS/SOL 14
Figure S2. Association of PRS-CRP and MRS-CRP with blood CRP level in MESA. 15
Figure S3. Correlation plot between log-transformed blood CRP level and various MRS-CRPs from major EWAS studies in HCHS/SOL 16
Figure S4. Forest plot comparing effect size of the MRS-CRP associations obtained using model 1 and model 2 in HCHS/SOL. 17
Figure S5. Forest plot showing associations of obstructive sleep apnea associated traits with diabetes, hypertension, and cognitive scores with and without adjusting for MRS-CRP in HCHS/SOL. 18
Figure S6. Forest plot comparing associations between MRS-CRP and health outcomes with and without adjusting for PRS-CRP in HCHS/SOL. 19
Figure S7. Cubic spline model fit plot for MRS-CRP and blood CRP with sleep duration in HCHS/SOL 20

Figure S8. Forest plot showing associations between blood and PRS-CRP and health outcomes using all available HCHS/SOL participants 21
Figure S9. Forest plot showing associations between CRP measures and measured health outcomes using participants with blood CRP level less than 10mg/L 22
Figure S10. Forest plot showing associations between CRP measures and measured health outcomes adjusting for physical activity levels, cigarette and alcohol use 23
Figure S11. Forest plot comparing effect size of the MRS-CRP associations of model 1 and sensitivity analyses in HCHS/SOL. 24
Supplementary References..25

Supplementary Methods: PRS Development

The Multi-Ethnic Study of Atherosclerosis (MESA)

MESA is a multi-center longitudinal cohort study comprising 6,814 men and women aged 45–84 years at baseline, self-identified from four race/ethnic groups without overt clinical CVD at the time of enrollment (Exam 1; 2000-2002)[1]. The current study included all participants with CRP measured at Exam 1 (N=6,415) with appropriate consent and excluded N=163 individuals without genetic data in the current dataset.

Assessment of CRP and Demographic Characteristics

High sensitivity CRP was measured during MESA Exam 1 using a BNII nephelometer with particle-enhanced immunonephelometric assays (Dade Behring Inc, Deerfield, IL). Sociodemographic characteristics, including age, sex and medical history, were collected using standard self-reported questionnaires. To report demographic characteristics, participants were stratified by clinical CRP risk groups for CVD (< 1mg/L [low risk], 1–<3 mg/L [Borderline], and ≥ 3mg/L [elevated risk])[2].

Whole-genome sequencing (WGS) and PRS development.

MESA WGS data was obtained from the Trans-Omics in Precision Medicine (TOPMed) program Freeze 10 release, with reads aligned to the human-genome build GRCh38 by a common pipeline across all sequencing centers[3]. Variant and genotype calling was performed jointly on all samples in a given TOPMed freeze[3]. Minor allele frequency (MAF) threshold for variant inclusion was set to MAF > 0.01 (computed over MESA participants). Genetic PCs and genetic relatedness was computed centrally by TOPMed using the PC-Relate and PC-AiR algorithms[4].

Multiple methods were employed to develop and construct genome-wide association analysis (GWAS)-derived PRS-CRP using publicly available summary statistics from UK Biobank (UKBB)[5] and Biobank Japan (BBJ)[6] for MESA and HCHS/SOL participants. UKBB data were divided into sets representing different genetic backgrounds: AFR (African, n = 6203), AMR (Amerindian, n = 937), EAS (East Asian, n = 2564), EUR (European, n = 400,094), and SAS (South Asian, n = 8397)[5].

First, UKBB-EAS and BBJ data were meta-analyzed using GWAMA (Genome-Wide Association Meta-Analysis) software[7] with default parameters allowing indel alleles. The GWAMA output, together with other UKBB ancestry-specific GWAS summary statistics, was then submitted to PRS-CSx[8] for development of variant weights. In PRS-CSx, variants present in at least one of the UKBB linkage disequilibrium reference panels were used to develop posterior effect estimates for each of the five genetic backgrounds[8], based on which PRSs were constructed using PRSice2[9] without clumping or thresholding. We also developed PRSs using PRSice2, based on UKBB+BBJ meta-analyzed GWAS summary statistics (combining all genetically-defined population groups), with clumping and thresholding set to $R^2 = 0.1$, distance = 1000 Kb, and p-value = 5×10^{-8}, 1×10^{-7}, 1×10^{-6}, 1×10^{-5}, 1×10^{-4}, 1×10^{-3}, 1×10^{-2}, 0.1.

A simple PRS summing the effect estimates for 48 genome-wide significant alleles associated with CRP[10] (referred to as Huang et al. PRS) was also constructed using PLINK[11]. For

comparison purposes, each calculated PRS was standardized by subtracting its mean and divided by its standard deviation.

PRS association analysis with CRP levels in MESA.

Association between each PRS-CRP and blood CRP level was assessed using simple linear regression in MESA, where blood CRP was log transformed to account for the right skewed distribution while adjusting for age, sex, body mass index (BMI), race, study site and first 5 genetic principal components (PCs). The best-performing PRS-CRP was determined as the one explaining highest variance in blood CRP levels and used in subsequent analysis in HCHS/SOL. We also carried forward a PRS developed as a weighted summation of PRS-CSx PRSs with weights determined by the regression coefficients from CRP regressed over all ancestry-specific PRS-CSx PRSs in MESA, where we further used either all MESA individuals, or only MESA Hispanic individuals. We do not report the results from association analyses of this PRS in MESA due to overfitting (as weights are developed based on analysis in MESA), but it was used in HCHS/SOL based on past work that demonstrated that such a weighted sum tends to outperform other PRSs[12].

Supplementary Tables

Table S1. Demographic characteristics of MESA

	**Overall** (n = 6411)	**Low risk (<1)** (n = 1926)	**Borderline (1-3)** (n = 2184)	**Elevated risk (>3)** (n = 2301)
Age (Mean (SD))	62.2 (10.2)	61.5 (10.7)	62.9 (10.2)	62.3 (9.8)
BMI (Mean (SD))	28.3 (5.5)	25.6 (4.2)	28.0 (4.6)	30.8 (6.0)
CRP (mg/L) (Mean (SD))	3.8 (5.9)	0.6 (0.2)	1.8 (0.6)	8.3 (8.0)
Gender (%) - Female	52.4	42	48	65.1
**Race/ethnicity (%)**				
White	39.3	41.2	39.9	37.1
Black	26	20.2	24.6	32.3
Chinese	12.1	22.2	11.5	4.2
Hispanic/Latino	22.6	16.3	24	26.4

Table S2. SNP counts in polygenic risk score (PRS) construction for MESA

Threshold/ Type	R2	Distance (Kb)	# SNP
BBJ+UKBB			
5.00E-08	0.1	1000	1162
1.00E-07			1249
1.00E-06			1760
1.00E-05			2956
1.00E-04			6186
1.00E-03			17126
1.00E-02			57357
1.00E-01			197806
PRS_CSx			
AFR	/	/	1165122
EUR			1055663
AMR			1119543
PRS_Huang et.al			
Weighted sum	/	/	48

Table S3. Association analysis for polygenic risk score (PRS)-CRP and blood CRP level in MESA

PRS Threshold/type	PRS effect estimate	SE	p value	Variance Explained (%)
**BBJ + UKBB, with clumping**				
**5.00E-08**	0.24	0.01	2.2E-73	5.38
**1.00E-07**	0.24	0.01	1.3E-70	5.63
**1.00E-06**	0.21	0.01	2.3E-50	4.48
**1.00E-05**	0.22	0.02	9.3E-23	3.64
**1.00E-04**	0.19	0.03	1.2E-09	2.65
**1.00E-03**	0.19	0.05	1.0E-04	2.32
**1.00E-02**	0.21	0.07	1.7E-03	2.2
**1.00E-01**	0.21	0.08	1.0E-02	2.16
**BBJ + UKBB, without clumping**				
**AFR**	0.03	0.01	1.8E-02	0.004
**EUR**	0.31	0.02	8.3E-83	9.33
**EAS**	0.1	0.01	3.54E-15	1.73
**SAS**	0.09	0.02	1.61E-08	0.7
**AMR**	0.002	0.02	8.7E-01	0.21
**48 SNPs from Huang et al.**				
**PRS_Huang**	0.25	0.01	6.2E-77	6.0

Table S4. Polygenic risk score (PRS) effect size obtained using MESA cohort

PRS-CSx	Effect estimate	SE	p value	Group
**AFR**	0.031	0.014	0.03	All MESA
**EUR**	0.288	0.016	5.11E-69	All MESA
**AMR**	0.001	0.015	0.93	All MESA
**EAS**	0.05	0.013	0.00012	All MESA
**SAS**	0.023	0.015	0.14	All MESA
**AFR**	0.042	0.028	0.13	MESA Hispanic
**EUR**	0.267	0.032	4.24E-16	MESA Hispanic
**AMR**	-0.007	0.029	0.79	MESA Hispanic
**EAS**	0.078	0.026	0.003	MESA Hispanic
**SAS**	0.061	0.029	0.038	MESA Hispanic

Effect sizes were obtained in a joint regression analysis of including all PRSs in the same regression model.

Table S5. SNP counts in ancestry-specific PRS construction in HCHS/SOL

Ancestry PRS	R2	Distance (Kb)	# SNP
PRS-CSx			
AFR	/	/	333979
EAS			302719
EUR			320917
AMR			330219
SAS			322661

Reduced number of SNPs compared to construction in MESA is due to different MAF and quality control filters in the HCHS/SOL imputed dataset.

Table S6. Background-specific effect estimates for MRS-CRP and PRS-CRP in HCHS/SOL

Background	Estimate	SE	Sample size	CRP Measure
Central American	0.38	0.058	214	MRS-CRP
Cuban	0.33	0.044	380	MRS-CRP
Dominican	0.48	0.061	241	MRS-CRP
Mexican	0.33	0.035	754	MRS-CRP
More than one/other heritage	0.38	0.14	41	MRS-CRP
Puerto Rican	0.41	0.041	432	MRS-CRP
South American	0.31	0.075	159	MRS-CRP
Central American	0.17	0.026	1296	PRS-CRP
Cuban	0.18	0.021	2009	PRS-CRP
Dominican	0.16	0.028	1177	PRS-CRP
Mexican	0.17	0.014	4503	PRS-CRP
More than one/other heritage	0.15	0.049	382	PRS-CRP
Puerto Rican	0.14	0.020	2149	PRS-CRP
South American	0.15	0.033	804	PRS-CRP

Cochran's heterogeneity test results: MRS-CRP (p-value = 0.33) and PRS-CRP (p-value = 0.8).
MRS-CRP: constructed based on an EWAS by Hillary *et al.* using elastic net regression; PRS-CRP: EUR: European ancestry-specific PRS-CSx.

Table S7. Association analysis for obstructive sleep apnea (OSA) cases with and without excessive daytime sleepiness (EDS) in HCHS/SOL

Comparison	PRS	Odds ratio	p value	95% CI	N
**MILD-TO-SEVERE OSA (REI>5)**					
OSA w/ EDS VS no OSA	PRS-ty	1.04	0.61	(0.89, 1.21)	8234
OSA w/o EDS VS no OSA	PRS-ty	0.98	0.55	(0.9, 1.06)	10274
All OSA VS no OSA	PRS-ty	0.99	0.86	(0.92, 1.07)	10956
OSA w/ EDS VS no OSA	PRS-EUR	0.88	0.05	(0.78, 1)	8234
OSA w/o EDS VS no OSA	PRS-EUR	0.96	0.3	(0.89, 1.04)	10274
All OSA VS no OSA	PRS-EUR	0.94	0.11	(0.88, 1.01)	10956
OSA w/ EDS VS no OSA	PRS-wsum	0.85	0.02	(0.75, 0.97)	8234
OSA w/o EDS VS no OSA	PRS-wsum	0.96	0.25	(0.89, 1.03)	10274
All OSA VS no OSA	PRS-wsum	0.94	0.07	(0.88, 1)	10956
**MODERATE-TO-SEVERE OSA (REI>15)**					
OSA w/ EDS VS Mild or no OSA	PRS-ty	1.26	0.04	(1.01, 1.56)	9956
OSA w/o EDS VS Mild or no OSA	PRS-ty	1.07	0.18	(0.97, 1.18)	10677
All OSA VS Mild or no OSA	PRS-ty	1.10	0.03	(1.01, 1.21)	10956
OSA w/ EDS VS Mild or no OSA	PRS-EUR	0.89	0.12	(0.76, 1.03)	9956
OSA w/o EDS VS Mild or no OSA	PRS-EUR	0.96	0.40	(0.86, 1.06)	10677
All OSA VS Mild or no OSA	PRS-EUR	0.94	0.15	(0.85, 1.02)	10956
OSA w/ EDS VS Mild or no OSA	PRS-wsum	0.88	0.10	(0.76, 1.02)	9956
OSA w/o EDS VS Mild or no OSA	PRS-wsum	0.95	0.38	(0.86, 1.06)	10677
All OSA VS Mild or no OSA	PRS-wsum	0.93	0.13	(0.85, 1.02)	10956

At the top part of the table OSA was defined according to REI>5 (i.e. mild-to-severe OSA, compared to no OSA), while at the bottom part of the table OSA was defined according to REI>15 (i.e. moderate-to-severe OSA, compared to mild or no OSA)

OSA: obstructive sleep apnea. w/: with. w/o: without. EDS: excessive daytime sleepiness. REI: respiratory event index.

PRS-ty: Huang et al. PRS. PRS-EUR: PRS-CSx of European ancestry. PRS-wsum: PRS-CSx with adaptive weights obtained using all MESA participants

Table S8. CpG sites selected by lasso penalized regression for respiratory event index

CPG	Coefficient	CHR	GENE NAME	Overlap
CG00574958	-0.39	11	CPT1A	Min SpO2, Diabetes
CG14476101	-0.67	1	PHGDH	Min SpO2, Hypertension
CG03246954	84.11	19	MKNK2	Min SpO2
CG14656297	-8.73	9	FXN	Min SpO2
CG23281327	-2.98	10		Min SpO2
CG23440058	-1.16	3	KALRN	Min SpO2
CG19693031	-0.02	1	TXNIP	Diabetes, Hypertension
CG00572560	-1.87	10		Diabetes
CG06690548	-0.43	4	SLC7A11	Hypertension
CG00241998	0.62	4	N4BP2	
CG00514616	2.63	7		
CG00607919	2.52	2	CCDC138	
CG01067983	-0.48	6	SERPINB6	
CG01112249	-7.51	10		
CG01526748	0.03	3	FGF12	
CG02622866	44.49	2	ATF2	
CG02761715	-1.12	12	DUSP16	
CG03292675	0.68	8	EPB49	
CG05132118	22.2	13		
CG05828624	-0.43	2	REG1A	
CG06076692	4.39	6	ATXN1	
CG07001630	32.1	2	TMEM214	
CG07453718	52.4	22	ARSA	
CG07794010	-0.53	1	SYT6	
CG07817279	7.87	17	SFRS1	
CG07884487	14.67	3	MITF	
CG07894567	-12.88	6		
CG08818130	3.86	3	ZNF654	
CG09048665	-10.84	16	WDR90	
CG10090326	-1.89	14	WDR25	
CG10322504	-4.57	20		
CG10365984	0.21	6	BACH2	
CG10370591	1.77	2	TPO	
CG10726559	-3.04	14	MIR127	
CG10759591	0.08	17	HRNBP3	
CG12008047	1.37	19	GDF15	
CG12090885	1.23	3	GSK3B	
CG12499235	-0.25	11	ASCL2	
CG13630239	1.2	10	RRP12	
CG13902024	-0.03	7	PLXNA4	
CG14037440	-0.91	9	FREM1	

CG14094164	1.01	13	
CG14532484	0.31	9	PAEP
CG14866339	-6.71	14	MIR379
CG14881305	-0.82	14	
CG15479387	0.18	6	LOC285830
CG15802555	-7.12	17	MPP2
CG16398761	-1.59	14	C14orf43
CG17087356	1.63	3	FHIT
CG17501395	1.21	6	ZC3H12D
CG17780956	2.89	4	MAP9
CG18262615	-15.02	3	KLHL6
CG19049696	-0.19	7	PKD1L1
CG19103704	0.49	19	FCGBP
CG19303434	9.93	4	SLC30A9
CG19756663	4.59	5	
CG19774973	-0.49	8	
CG20644120	15.49	12	SPSB2
CG20820543	-14.21	6	MYB
CG21877216	2.6	6	GNL1
CG22980156	-0.49	17	CDRT4
CG25285658	0.3	4	MRFAP1L1
CG26104690	-6.04	7	PRKAR2B
CG26800884	-1.85	4	KLB
CG27255239	7.84	2	RFX8
CG27262415	2.82	11	PTPRCAP

Table S9. CpG sites selected by Lasso penalized regression for minimum oxygen saturation

CPG	Coefficient	CHR	GENE NAME	Overlap
CG00574958	0.08	11	CPT1A	OSA, Diabetes
CG14476101	0	1	PHGDH	OSA, Hypertension
CG03246954	-0.22	19	MKNK2	OSA
CG14656297	0.08	9	FXN	OSA
CG23281327	0.15	10		OSA
CG23440058	0.05	3	KALRN	OSA
CG00816397	-0.76	1	PFDN2	
CG05802514	0.05	4		
CG09456254	0.01	21	C21orf130	
CG09728637	0.04	18	TYMS	
CG12450708	0.02	10		
CG24083756	-0.19	21	MRPS6	

Table S10. CpG sites selected by Lasso penalized regression for diabetes

CPG	Coefficient	CHR	GENE NAME
**CG00134210**	-18.535675	10	FAM107B
**CG00572560**	0.38729852	10	
**CG00574958**	-13.544056	11	CPT1A
**CG00972420**	-2.9686603	20	FAM65C
**CG01509330**	0.96493426	6	
**CG02298525**	-0.6004831	1	
**CG02481950**	1.02788524	16	IGSF6
**CG02576503**	-2.742051	19	SLC27A1
**CG02650017**	-8.2219207	17	PHOSPHO1
**CG02667103**	-0.1862179	12	TMTC2
**CG02855983**	4.43477074	1	PKP1
**CG03432464**	-0.0230474	15	SNORD116-2
**CG03601585**	4.33533405	11	SLC3A2
**CG03987653**	0.61893104	16	DEF8;DEF8
**CG04216117**	0.96327811	11	
**CG05815275**	-1.7289152	11	ANKK1
**CG06192883**	6.49855904	15	MYO5C
**CG06500161**	7.38178863	21	ABCG1
**CG07282465**	-4.4641372	8	PSD3;PSD3
**CG07300660**	1.74868506	2	GLI2
**CG08034990**	-0.4273852	2	GREB1
**CG09031958**	0.89751208	5	MAML1
**CG09505788**	0.23297997	7	
**CG09552517**	-1.2128646	15	SIN3A

**CG10288578**	0.1891789	2	FAM49A
**CG10513161**	3.31999286	3	ABCC5
**CG11024682**	2.6017879	17	SREBF1
**CG11111859**	1.47270172	10	C10orf131
**CG11404039**	-11.130853	4	PLRG1
**CG11523799**	0.86805843	6	YIPF3
**CG11607604**	-2.5366099	13	FARP1
**CG11647481**	1.0209757	7	FOXK1
**CG13274938**	0.04128576	17	RARA
**CG13469874**	0.12535074	4	CTBP1
**CG13982695**	-0.2356797	11	TUB
**CG14547801**	-0.5698695	19	PPFIA3
**CG14564724**	0.59909364	9	ABCA1
**CG14662283**	6.5679891	6	WDR27
**CG15357118**	1.77678106	2	UGGT1
**CG15523998**	-0.4725048	13	RNF17
**CG17470397**	-0.0741813	3	
**CG17636309**	0.29398983	8	EEF1D
**CG17827328**	6.49430799	1	STK40
**CG19459834**	-1.6601355	3	DHX36
**CG19693031**	-9.7856143	1	TXNIP
**CG20651995**	-2.2378118	2	
**CG21042285**	1.95146578	13	SACS
**CG21123686**	-4.6590825	5	
**CG22012981**	0.29473032	3	ACOX2
**CG22207405**	-1.7404525	2	
**CG23885932**	-16.805159	19	SBNO2
**CG25851745**	1.6737256	1	COL8A2
**CG25940949**	8.01663924	6	RGL2
**CG26393791**	-0.6702109	2	
**CG27247736**	-0.4885003	6	PNLDC1

Table S11. CpG sites selected by Lasso penalized regression for Hypertension

CPG	Coefficient	CHR	GENE NAME
**CG04010915**	0.42120254	12	
**CG06500161**	0.53102502	21	ABCG1
**CG06690548**	-0.2610127	4	SLC7A11
**CG09716038**	6.03627227	3	MINA
**CG14476101**	-0.0274912	1	PHGDH
**CG19693031**	-0.1062296	1	TXNIP

Table S12. One sample Mendelian Randomization analysis for blood CRP and MRS-CRP as potential causes

Trait (outcome)	CRP measure (exposure)	Instrument	Causal effect estimate	p-value
REI	Blood CRP	PRS-EUR	-0.13	0.84
Min SPO2	Blood CRP	PRS-EUR	0.29	0.37
Mean SPO2	Blood CRP	PRS-EUR	0.02	0.7
% SPO2	Blood CRP	PRS-EUR	-0.13	0.46
WHIIRS	Blood CRP	PRS-EUR	-0.02	0.96
ESS	Blood CRP	PRS-EUR	-0.46	0.08
SLPDUR	Blood CRP	PRS-EUR	0.08	0.31
REI	Blood CRP	PRS-wsum-all	-0.38	0.53
Min SPO2	Blood CRP	PRS-wsum-all	0.28	0.36
Mean SPO2	Blood CRP	PRS-wsum-all	0.03	0.54
% SPO2	Blood CRP	PRS-wsum-all	-0.18	0.3
WHIIRS	Blood CRP	PRS-wsum-all	-0.06	0.84
ESS	Blood CRP	PRS-wsum-all	-0.49	0.05
SLPDUR	Blood CRP	PRS- wsum-all	0.08	0.28
REI	MRS-CRP	PRS-EUR	2.45	0.66
Min SPO2	MRS-CRP	PRS-EUR	0.35	0.91
Mean SPO2	MRS-CRP	PRS-EUR	0.29	0.51
% SPO2	MRS-CRP	PRS-EUR	-0.8	0.59
WHIIRS	MRS-CRP	PRS-EUR	1.16	0.69
ESS	MRS-CRP	PRS-EUR	0.66	0.8
SLPDUR	MRS-CRP	PRS-EUR	0.7	0.34
REI	MRS-CRP	PRS-wsum-all	1.4	0.78
Min SPO2	MRS-CRP	PRS-wsum-all	-0.05	0.98
Mean SPO2	MRS-CRP	PRS-wsum-all	0.3	0.46
% SPO2	MRS-CRP	PRS-wsum-all	-0.97	0.48
WHIIRS	MRS-CRP	PRS-wsum-all	0.86	0.74
ESS	MRS-CRP	PRS-wsum-all	0.68	0.78
SLPDUR	MRS-CRP	PRS-wsum-all	0.67	0.32

REI: Respiratory event index; **Min SpO2**: minimum oxygen saturation; **Mean SpO2**: mean oxygen saturation; **% SpO2**: percentage of total monitoring time where oxygen saturation is below 90%; **WHIIRS**: Women's Health Initiative Insomnia Rating Scale; **ESS**: Epworth Sleepiness Scale; **SLPDUR**: sleep duration (modelled linearly)

Supplementary Figures

Figure S1. Density plots for original and log-transformed blood CRP levels in HCHS/SOL

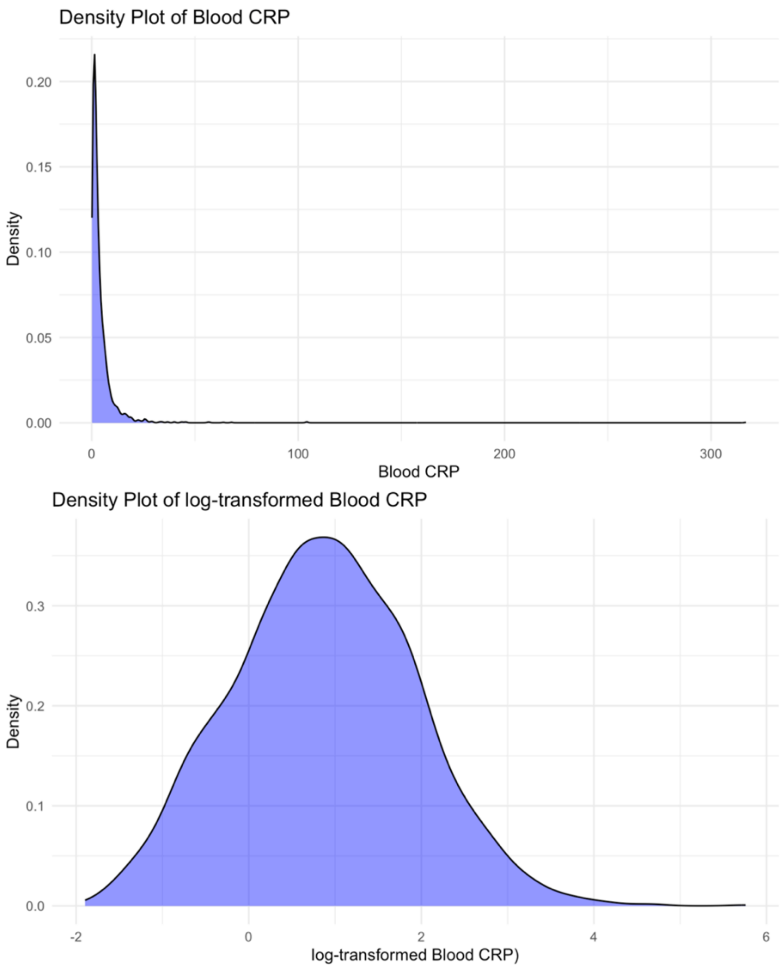

Figure S2. Association of PRS-CRP and MRS-CRP with blood CRP level in MESA.

Association	Coefficient (95% CI)	p-value
**MRS-CRP**	0.36 (0.31 - 0.42)	9.5e-33
**PRS-EUR**	0.20 (0.15 - 0.25)	2.2e-14
**PRS-wsum-All**	0.21 (0.16 - 0.26)	5.7e-15
**PRS-wsum-Hispanic**	0.21 (0.16 - 0.26)	1.6e-14

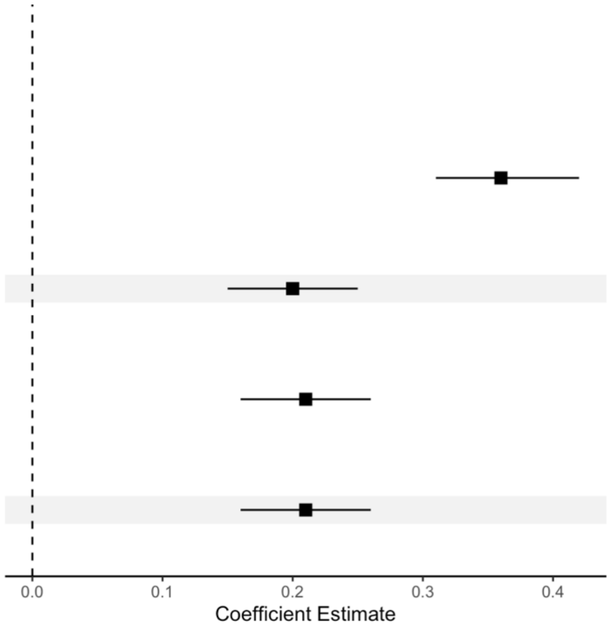

EUR: European ancestry; PRS-wsum-All: PRS calculated as weighted sum of PRSs-CSx with weights obtained using all MESA participants; PRS-wsum-Hispanic: PRS calculated as weighted sum of PRSs-CSx with weights obtained using all Hispanic participants

Figure S3. Correlation plot between log-transformed blood CRP level and various MRS-CRPs from major EWAS studies in HCHS/SOL

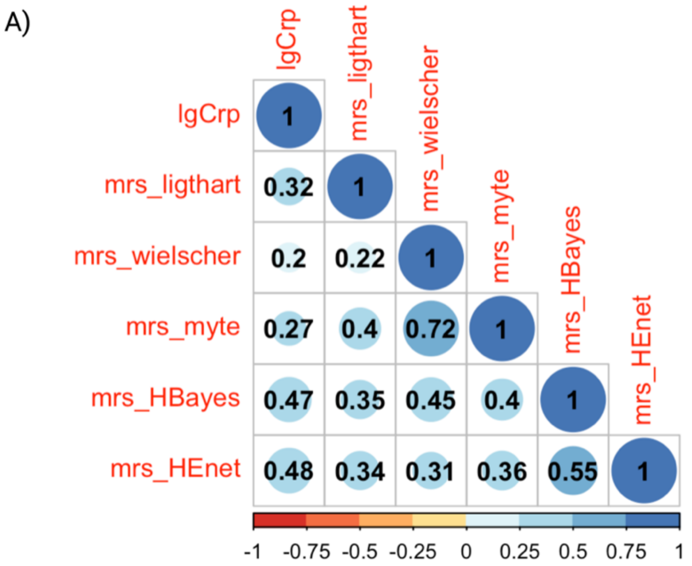

lgCrp: log-transformed blood CRP level; mrs_ligthart: (MRS-CRP based on an EWAS by Ligthart *et al.*); mrs_wielscher: (MRS-CRP based on an EWAS by Wielscher *et al.*); mrs_myte: (MRS-CRP based on a replication study by Myte *et al.*); mrs_HBayes and mrs HEnet (MRS-CRPs based on an EWAS by Hillary *et al.* using Bayesian penalised regression and elastic net regression, respectively)

Figure S4. Forest plot comparing effect size of the MRS-CRP associations obtained using model 1 and model 2 in HCHS/SOL.

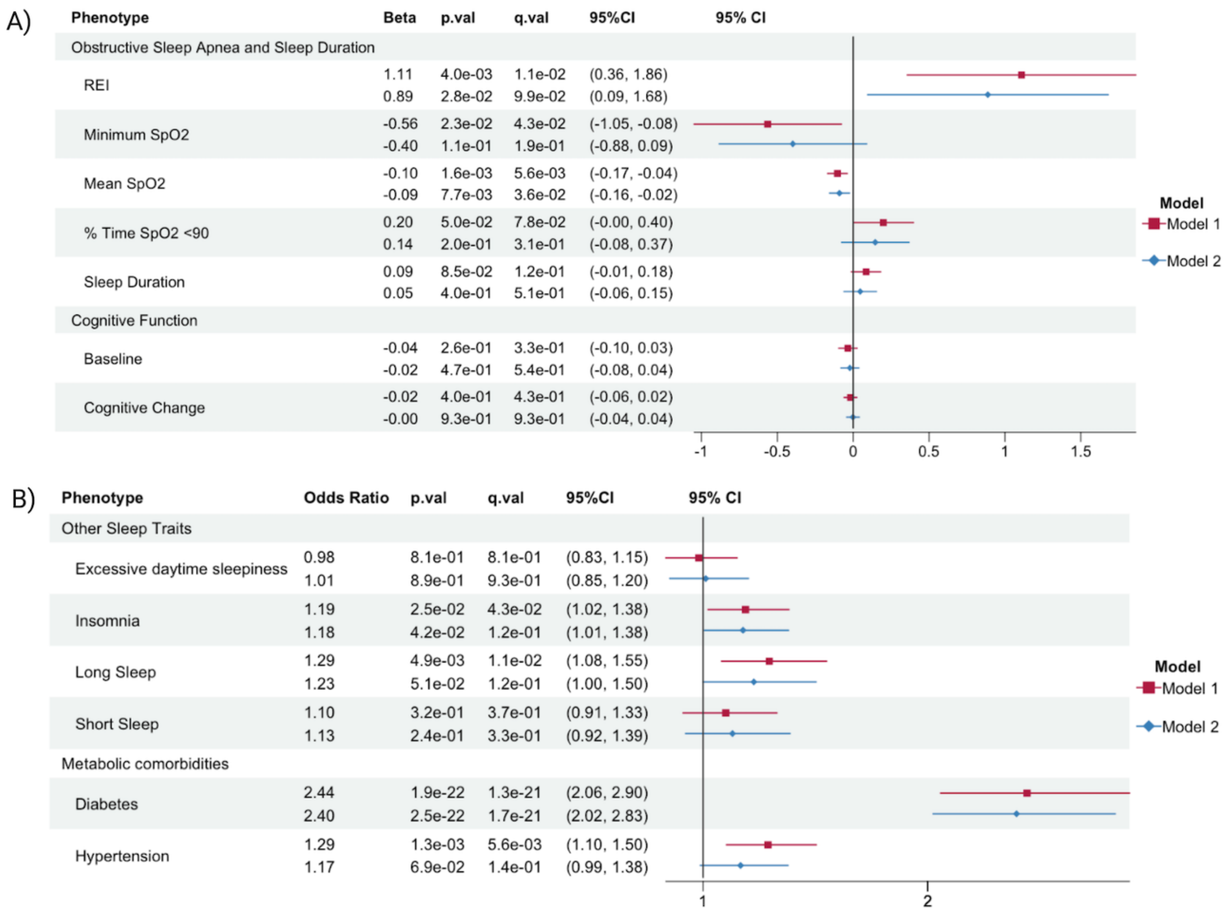

A) obstructive sleep apnea (OSA), sleep duration and cognitive traits; B) odds ratio for binary cardio-metabolic and other sleep outcomes. From left to right model coefficients or odds ratio in case of binary outcomes, p value (p.val), FDR corrected q value (q.val) and 95% confidence interval (95% CI). REI: respiratory event index; SpO2: oxygen saturation; Cognitive function: change in cognitive function score between baseline and SOL-INCA visit (Change); cognitive function score at baseline (Baseline). Model 1 covariates: age, sex, BMI, study center, Hispanic/Latino background, and first 5 genetic principal components. Model 2 covariates: age, sex, BMI, study center, Hispanic/Latino background, first 5 genetic principal components, diabetes and hypertension status.

Figure S5. Forest plot showing associations of obstructive sleep apnea associated traits with diabetes, hypertension, and cognitive scores with and without adjusting for MRS-CRP in HCHS/SOL.

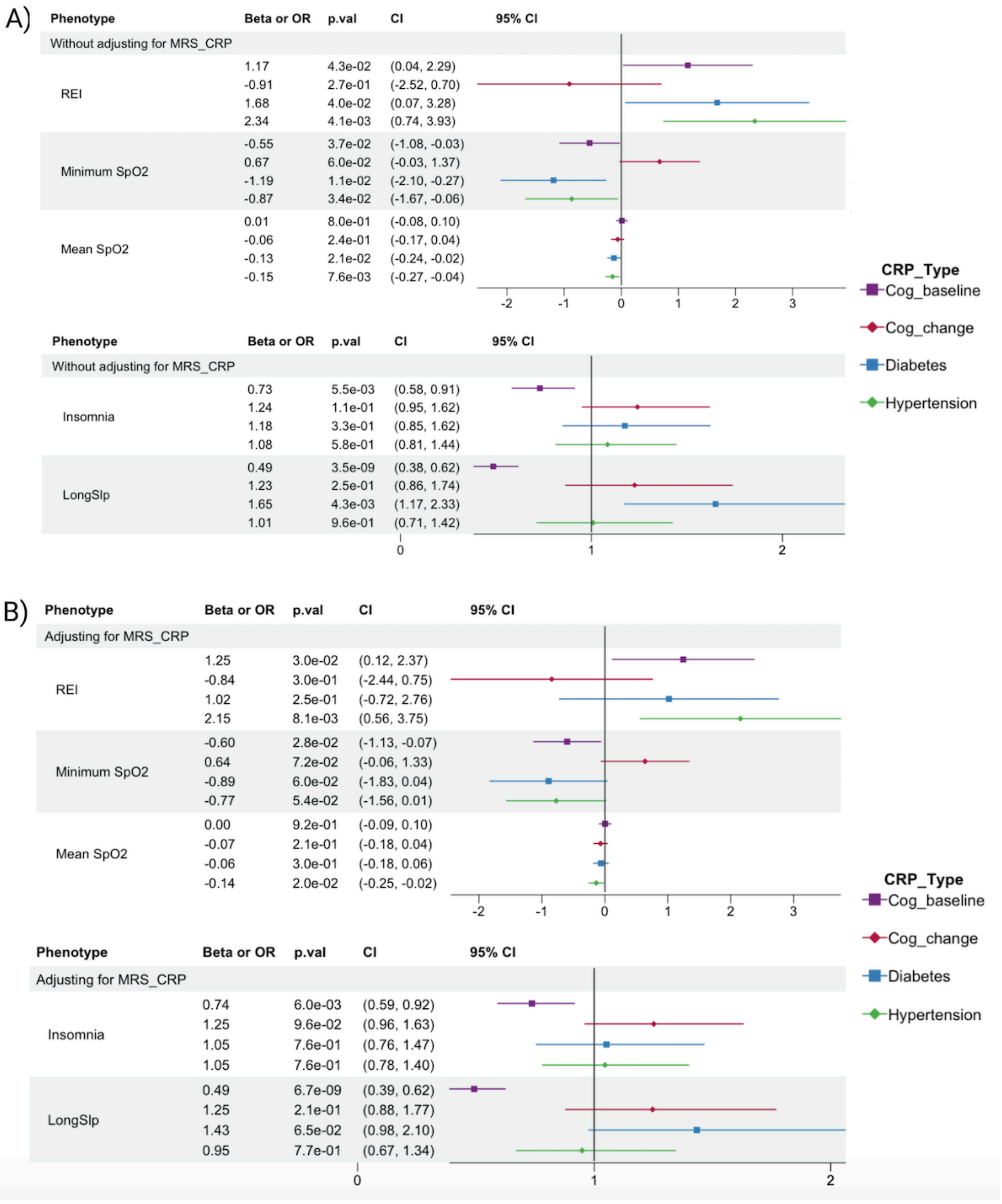

A) Association results without adjusting for MRS-CRP; B) Association results with MRS-CRP adjusted as covariate in the model. From left to right model coefficients (Beta or OR: odds ratio), p value (p.val) and 95% confidence interval (CI). REI: respiratory event index; SpO2: oxygen saturation; Cog_baseline: cognitive function score at baseline; Cog_change: change in cognitive function score between baseline and SOL-INCA visit; LongSlp: long sleep (sleep duration > 9 hours). Covariates: age, sex, BMI, study sites, Hispanic/Latino background, and first 5 genetic principal components.

Figure S6. Forest plot comparing associations between MRS-CRP and health outcomes with and without adjusting for PRS-CRP in HCHS/SOL.

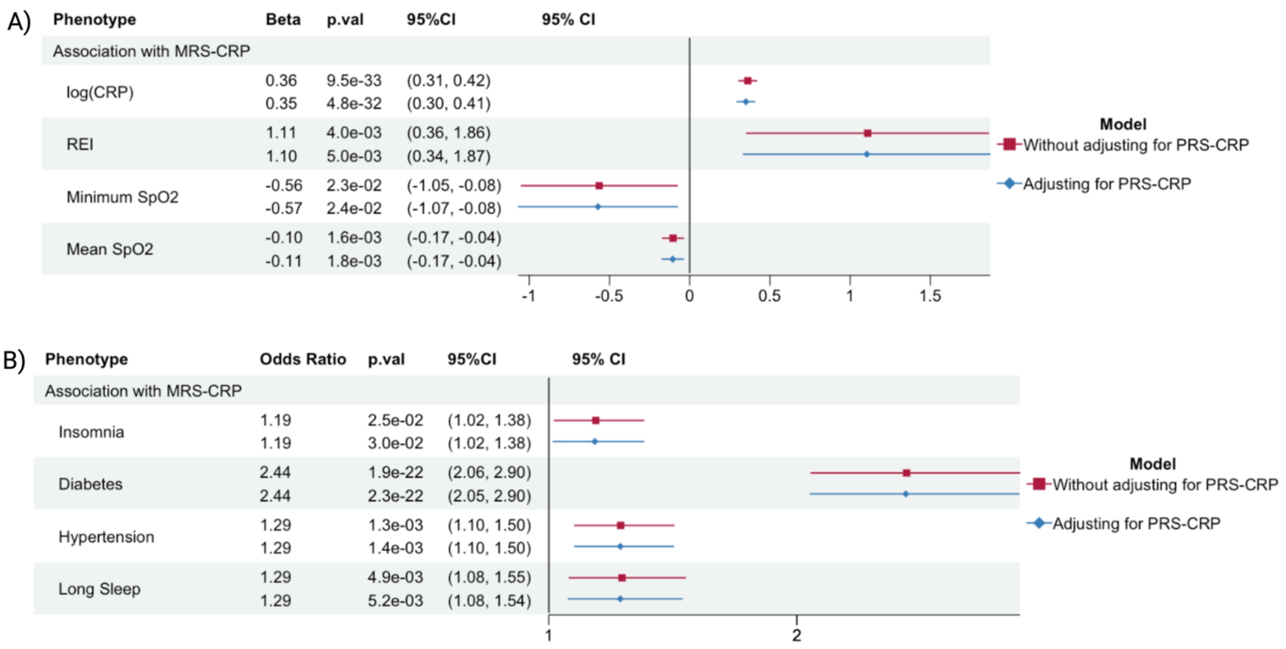

A) Association between MRS-CRP and listed phenotypes modeled as continuous variables; B) Association between MRS-CRP and listed phenotypes modeled as binary variables. From left to right model coefficients or odds ratio in case of binary outcomes, p value (p.val) and 95% confidence interval (95% CI). Log(CRP): log-transformed blood CRP level; REI: respiratory event index; SpO2: oxygen saturation.

Figure S7. Cubic spline model fit plot for MRS-CRP and blood CRP with sleep duration in HCHS/SOL

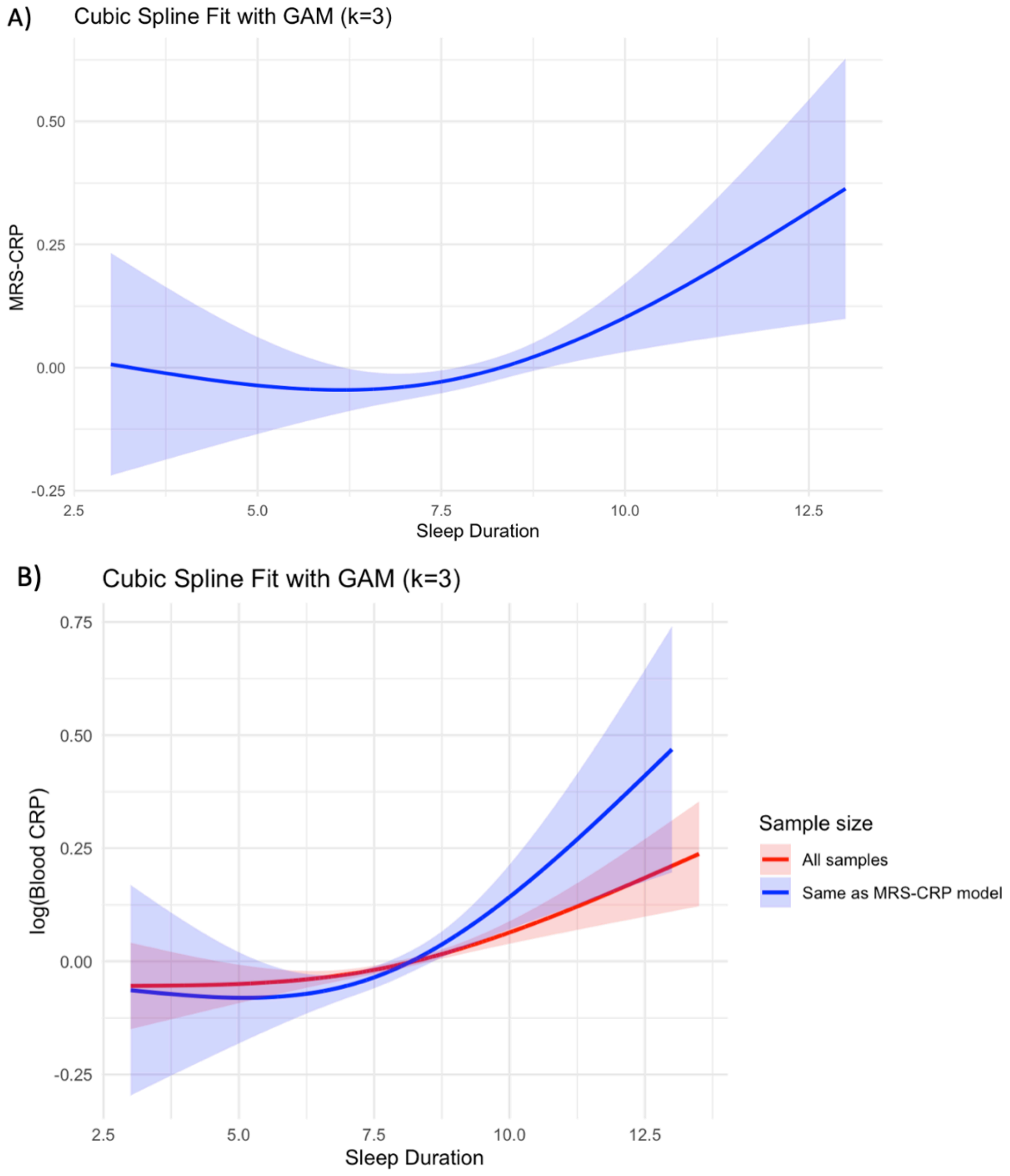

A) Cubic spline model fit for MRS-CRP; B) Cubic spline model fit for blood CRP level using all participants and same participants as MRS-CRP model.
Sleep Duration is measured in hours.

Figure S8. Forest plot showing associations between blood and PRS-CRP and health outcomes using all available HCHS/SOL participants

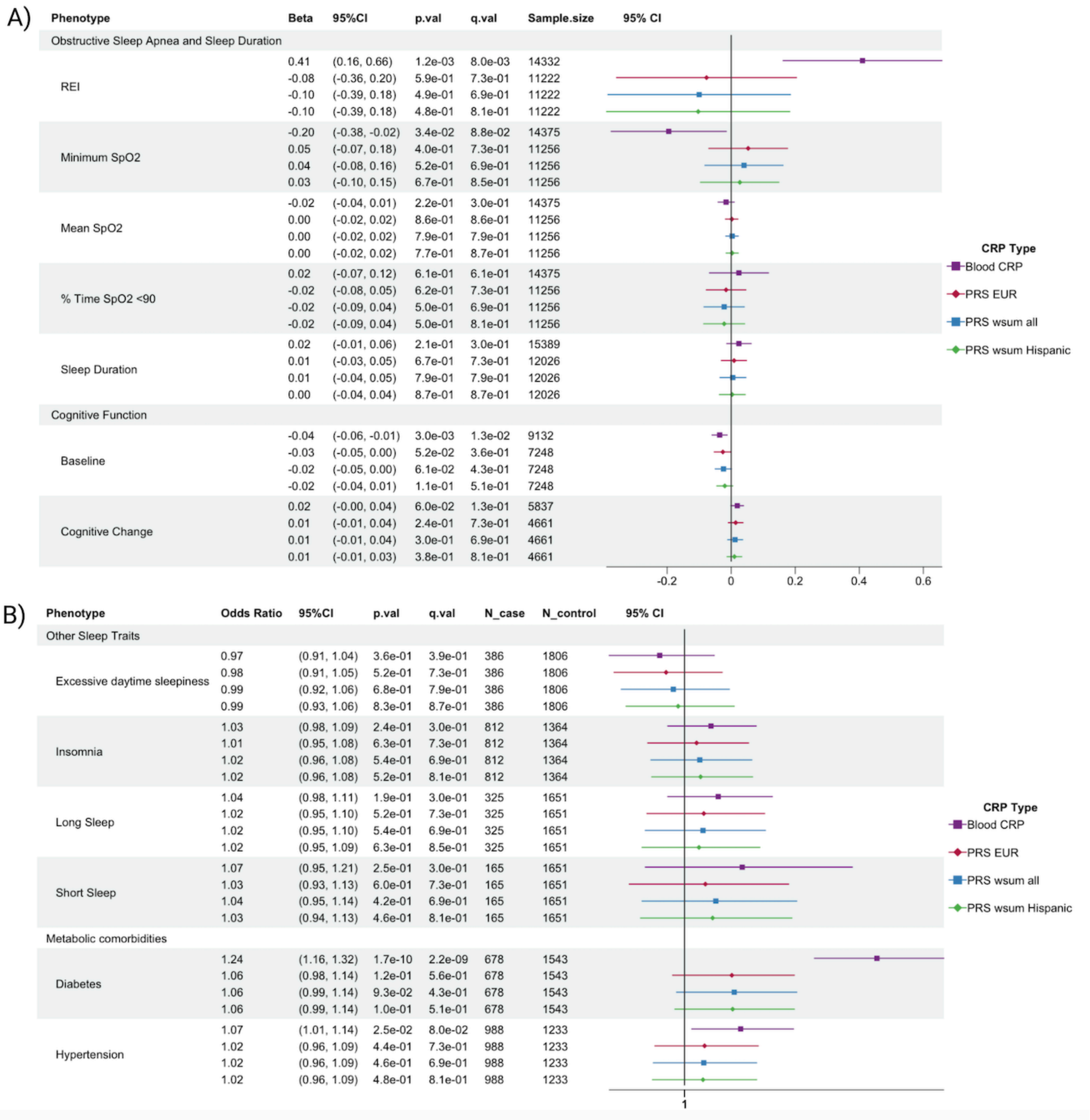

A)

Phenotype	Beta	95%CI	p.val	q.val	Sample.size
**Obstructive Sleep Apnea and Sleep Duration**					
REI	0.41	(0.16, 0.66)	1.2e-03	8.0e-03	14332
	-0.08	(-0.36, 0.20)	5.9e-01	7.3e-01	11222
	-0.10	(-0.39, 0.18)	4.9e-01	6.9e-01	11222
	-0.10	(-0.39, 0.18)	4.8e-01	8.1e-01	11222
Minimum SpO2	-0.20	(-0.38, -0.02)	3.4e-02	8.8e-02	14375
	0.05	(-0.07, 0.18)	4.0e-01	7.3e-01	11256
	0.04	(-0.08, 0.16)	5.2e-01	6.9e-01	11256
	0.03	(-0.10, 0.15)	6.7e-01	8.5e-01	11256
Mean SpO2	-0.02	(-0.04, 0.01)	2.2e-01	3.0e-01	14375
	0.00	(-0.02, 0.02)	8.6e-01	8.6e-01	11256
	0.00	(-0.02, 0.02)	7.9e-01	7.9e-01	11256
	0.00	(-0.02, 0.02)	7.7e-01	8.7e-01	11256
% Time SpO2 <90	0.02	(-0.07, 0.12)	6.1e-01	6.1e-01	14375
	-0.02	(-0.08, 0.05)	6.2e-01	7.3e-01	11256
	-0.02	(-0.09, 0.04)	5.0e-01	6.9e-01	11256
	-0.02	(-0.09, 0.04)	5.0e-01	8.1e-01	11256
Sleep Duration	0.02	(-0.01, 0.06)	2.1e-01	3.0e-01	15389
	0.01	(-0.03, 0.05)	6.7e-01	7.3e-01	12026
	0.01	(-0.04, 0.05)	7.9e-01	7.9e-01	12026
	0.00	(-0.04, 0.04)	8.7e-01	8.7e-01	12026
**Cognitive Function**					
Baseline	-0.04	(-0.06, -0.01)	3.0e-03	1.3e-02	9132
	-0.03	(-0.05, 0.00)	5.2e-02	3.6e-01	7248
	-0.02	(-0.05, 0.00)	6.1e-02	4.3e-01	7248
	-0.02	(-0.04, 0.01)	1.1e-01	5.1e-01	7248
Cognitive Change	0.02	(-0.00, 0.04)	6.0e-02	1.3e-01	5837
	0.01	(-0.01, 0.04)	2.4e-01	7.3e-01	4661
	0.01	(-0.01, 0.04)	3.0e-01	6.9e-01	4661
	0.01	(-0.01, 0.03)	3.8e-01	8.1e-01	4661

B)

Phenotype	Odds Ratio	95%CI	p.val	q.val	N_case	N_control
**Other Sleep Traits**						
Excessive daytime sleepiness	0.97	(0.91, 1.04)	3.6e-01	3.9e-01	386	1806
	0.98	(0.91, 1.05)	5.2e-01	7.3e-01	386	1806
	0.99	(0.92, 1.06)	6.8e-01	7.9e-01	386	1806
	0.99	(0.93, 1.06)	8.3e-01	8.7e-01	386	1806
Insomnia	1.03	(0.98, 1.09)	2.4e-01	3.0e-01	812	1364
	1.01	(0.95, 1.08)	6.3e-01	7.3e-01	812	1364
	1.02	(0.96, 1.08)	5.4e-01	6.9e-01	812	1364
	1.02	(0.96, 1.08)	5.2e-01	8.1e-01	812	1364
Long Sleep	1.04	(0.98, 1.11)	1.9e-01	3.0e-01	325	1651
	1.02	(0.95, 1.10)	5.2e-01	7.3e-01	325	1651
	1.02	(0.95, 1.10)	5.4e-01	6.9e-01	325	1651
	1.02	(0.95, 1.09)	6.3e-01	8.5e-01	325	1651
Short Sleep	1.07	(0.95, 1.21)	2.5e-01	3.0e-01	165	1651
	1.03	(0.93, 1.13)	6.0e-01	7.3e-01	165	1651
	1.04	(0.95, 1.14)	4.2e-01	6.9e-01	165	1651
	1.03	(0.94, 1.13)	4.6e-01	8.1e-01	165	1651
**Metabolic comorbidities**						
Diabetes	1.24	(1.16, 1.32)	1.7e-10	2.2e-09	678	1543
	1.06	(0.98, 1.14)	1.2e-01	5.6e-01	678	1543
	1.06	(0.99, 1.14)	9.3e-02	4.3e-01	678	1543
	1.06	(0.99, 1.14)	1.0e-01	5.1e-01	678	1543
Hypertension	1.07	(1.01, 1.14)	2.5e-02	8.0e-02	988	1233
	1.02	(0.96, 1.09)	4.4e-01	7.3e-01	988	1233
	1.02	(0.96, 1.09)	4.6e-01	6.9e-01	988	1233
	1.02	(0.96, 1.09)	4.8e-01	8.1e-01	988	1233

A) obstructive sleep apnea (OSA), sleep duration and cognitive traits; B) odds ratio for binary cardio-metabolic and other sleep outcomes. From left to right model coefficients or odds ratio in case of binary outcomes (Beta), 95% confidence interval (95% CI), p value (p.val), FDR corrected q value (q.val) and sample size.

Figure S9. Forest plot showing associations between CRP measures and measured health outcomes using participants with blood CRP level less than 10mg/L

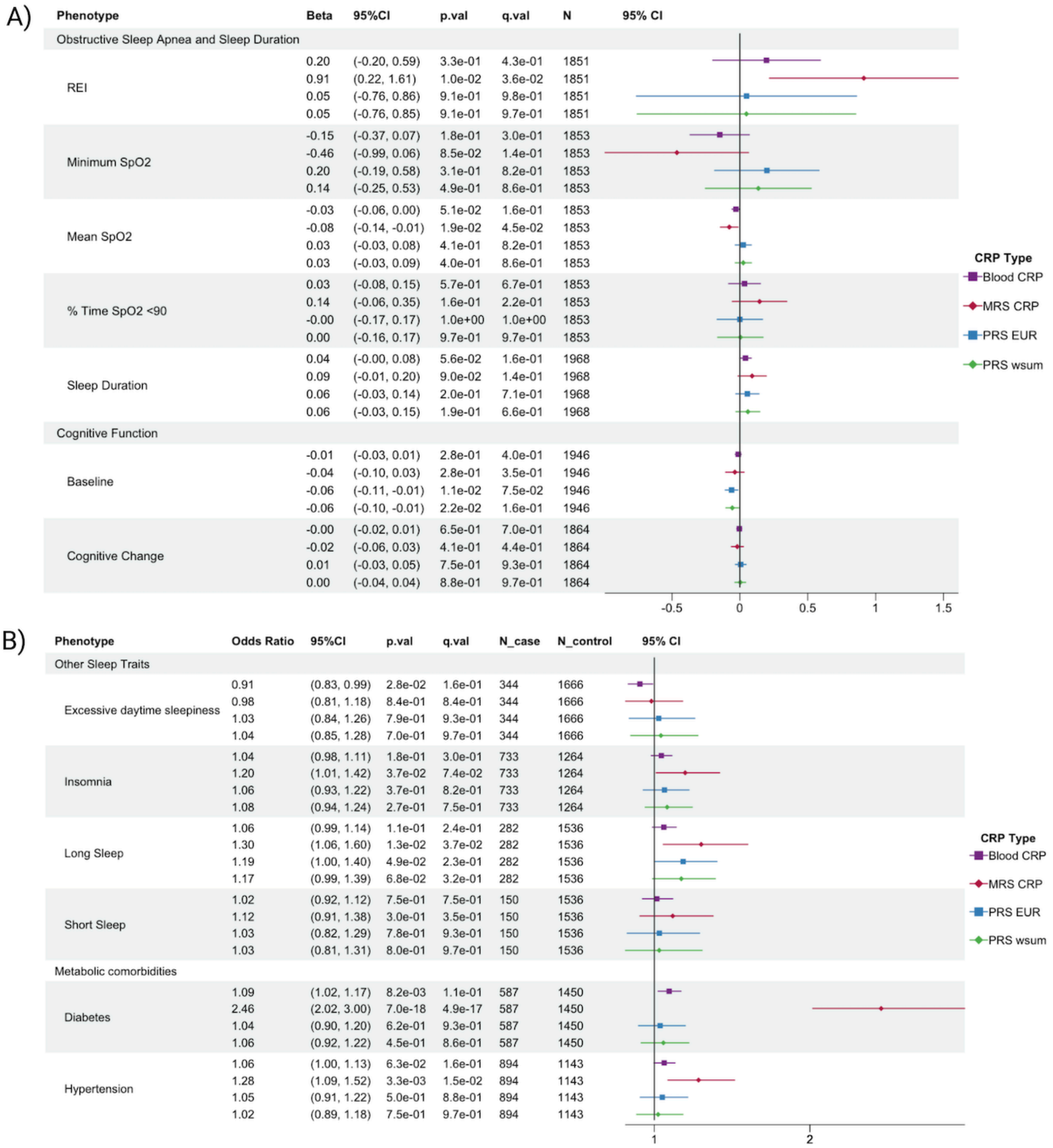

A) obstructive sleep apnea (OSA), sleep duration and cognitive traits; B) odds ratio for binary cardio-metabolic and other sleep outcomes. From left to right model coefficients or odds ratio in case of binary outcomes, p value (p.val), FDR corrected q value (q.val) and 95% confidence interval (95% CI). REI: respiratory event index; SpO2: oxygen saturation; Cognitive function: change in cognitive function score between baseline and SOL-INCA visit (Change); cognitive function score at baseline (Baseline).

Figure S10. Forest plot showing associations between CRP measures and measured health outcomes adjusting for physical activity levels, cigarette and alcohol use

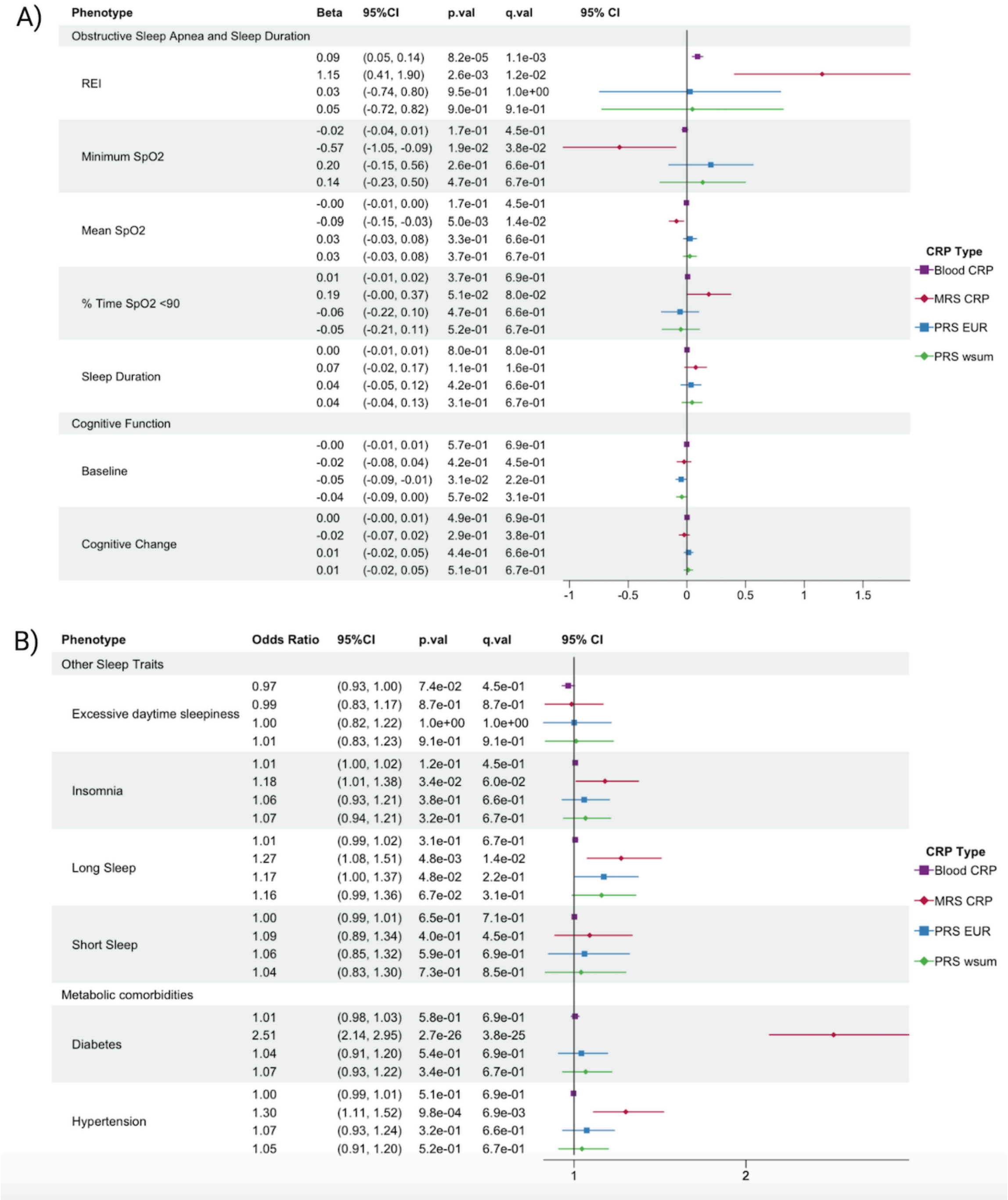

A) obstructive sleep apnea (OSA), sleep duration and cognitive traits; B) odds ratio for binary cardio-metabolic and other sleep outcomes. From left to right model coefficients or odds ratio in case of binary outcomes, p value (p.val), FDR corrected q value (q.val) and 95% confidence interval (95% CI). REI: respiratory event index; SpO2: oxygen saturation; Cognitive function: change in cognitive function score between baseline and SOL-INCA visit (Change); cognitive function score at baseline (Baseline).

Figure S11. Forest plot comparing effect size of the MRS-CRP associations of model 1 and sensitivity analyses in HCHS/SOL.

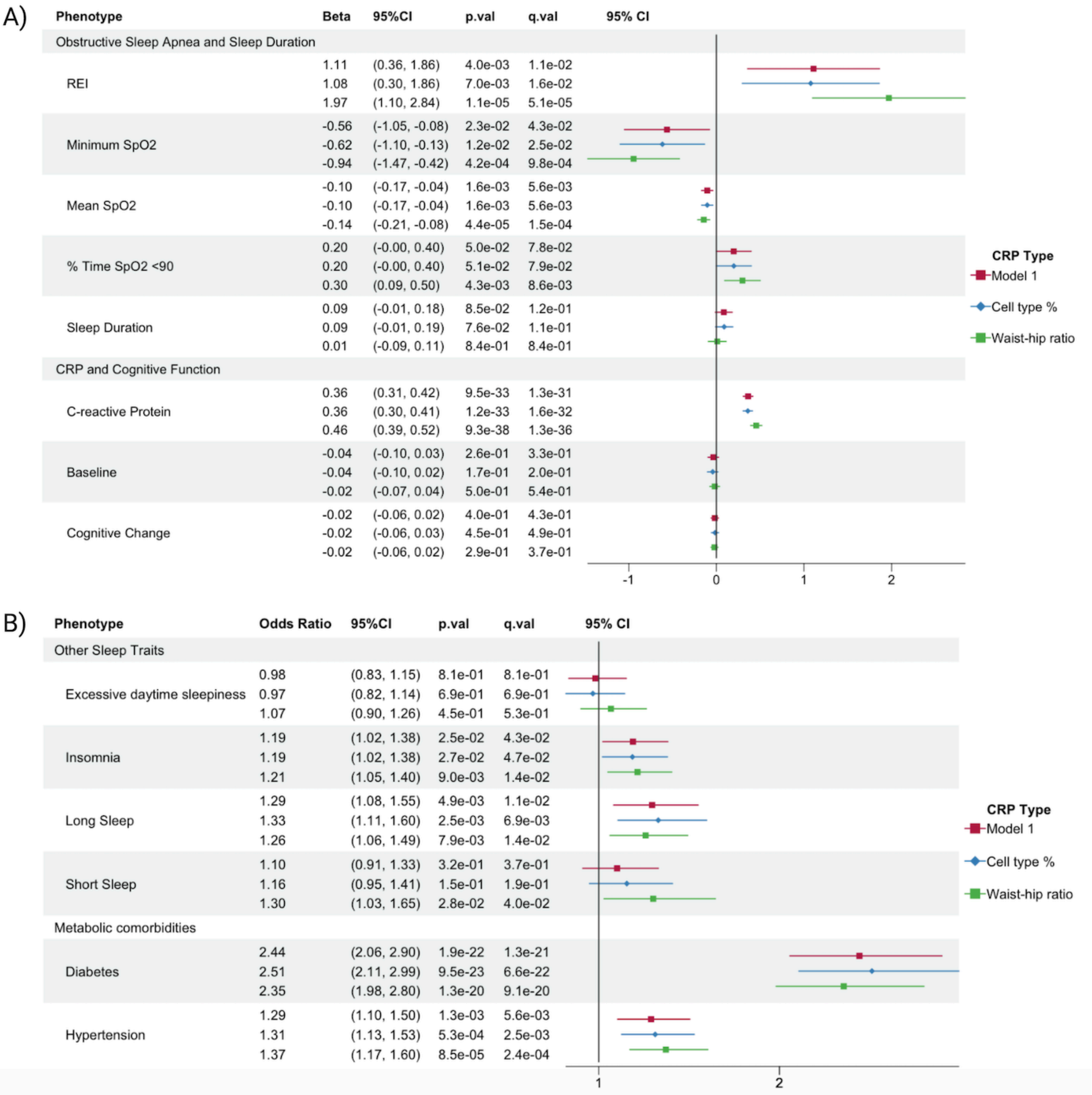

A)

Phenotype	Beta	95%CI	p.val	q.val
**Obstructive Sleep Apnea and Sleep Duration**				
REI	1.11	(0.36, 1.86)	4.0e-03	1.1e-02
	1.08	(0.30, 1.86)	7.0e-03	1.6e-02
	1.97	(1.10, 2.84)	1.1e-05	5.1e-05
Minimum SpO2	-0.56	(-1.05, -0.08)	2.3e-02	4.3e-02
	-0.62	(-1.10, -0.13)	1.2e-02	2.5e-02
	-0.94	(-1.47, -0.42)	4.2e-04	9.8e-04
Mean SpO2	-0.10	(-0.17, -0.04)	1.6e-03	5.6e-03
	-0.10	(-0.17, -0.04)	1.6e-03	5.6e-03
	-0.14	(-0.21, -0.08)	4.4e-05	1.5e-04
% Time SpO2 <90	0.20	(-0.00, 0.40)	5.0e-02	7.8e-02
	0.20	(-0.00, 0.40)	5.1e-02	7.9e-02
	0.30	(0.09, 0.50)	4.3e-03	8.6e-03
Sleep Duration	0.09	(-0.01, 0.18)	8.5e-02	1.2e-01
	0.09	(-0.01, 0.19)	7.6e-02	1.1e-01
	0.01	(-0.09, 0.11)	8.4e-01	8.4e-01
**CRP and Cognitive Function**				
C-reactive Protein	0.36	(0.31, 0.42)	9.5e-33	1.3e-31
	0.36	(0.30, 0.41)	1.2e-33	1.6e-32
	0.46	(0.39, 0.52)	9.3e-38	1.3e-36
Baseline	-0.04	(-0.10, 0.03)	2.6e-01	3.3e-01
	-0.04	(-0.10, 0.02)	1.7e-01	2.0e-01
	-0.02	(-0.07, 0.04)	5.0e-01	5.4e-01
Cognitive Change	-0.02	(-0.06, 0.02)	4.0e-01	4.3e-01
	-0.02	(-0.06, 0.03)	4.5e-01	4.9e-01
	-0.02	(-0.06, 0.02)	2.9e-01	3.7e-01

B)

Phenotype	Odds Ratio	95%CI	p.val	q.val
**Other Sleep Traits**				
Excessive daytime sleepiness	0.98	(0.83, 1.15)	8.1e-01	8.1e-01
	0.97	(0.82, 1.14)	6.9e-01	6.9e-01
	1.07	(0.90, 1.26)	4.5e-01	5.3e-01
Insomnia	1.19	(1.02, 1.38)	2.5e-02	4.3e-02
	1.19	(1.02, 1.38)	2.7e-02	4.7e-02
	1.21	(1.05, 1.40)	9.0e-03	1.4e-02
Long Sleep	1.29	(1.08, 1.55)	4.9e-03	1.1e-02
	1.33	(1.11, 1.60)	2.5e-03	6.9e-03
	1.26	(1.06, 1.49)	7.9e-03	1.4e-02
Short Sleep	1.10	(0.91, 1.33)	3.2e-01	3.7e-01
	1.16	(0.95, 1.41)	1.5e-01	1.9e-01
	1.30	(1.03, 1.65)	2.8e-02	4.0e-02
**Metabolic comorbidities**				
Diabetes	2.44	(2.06, 2.90)	1.9e-22	1.3e-21
	2.51	(2.11, 2.99)	9.5e-23	6.6e-22
	2.35	(1.98, 2.80)	1.3e-20	9.1e-20
Hypertension	1.29	(1.10, 1.50)	1.3e-03	5.6e-03
	1.31	(1.13, 1.53)	5.3e-04	2.5e-03
	1.37	(1.17, 1.60)	8.5e-05	2.4e-04

A) obstructive sleep apnea (OSA), sleep duration and cognitive traits; B) odds ratio for binary cardio-metabolic and other sleep outcomes. From left to right model coefficients or odds ratio in case of binary outcomes, p value (p.val), FDR corrected q value (q.val) and 95% confidence interval (95% CI). REI: Respiratory event index; SpO2: oxygen saturation; Cognitive function: change in cognitive function score between baseline and SOL-INCA visit (Change); cognitive function score at baseline (Baseline).

Supplementary References

1. Bild, D. E. *et al.* Multi-Ethnic Study of Atherosclerosis: Objectives and design. *Am J Epidemiol* **156**, 871–881 (2002).
2. Pearson, T. A. *et al.* Markers of Inflammation and Cardiovascular Disease. *Circulation* **107**, 499–511 (2003).
3. Taliun, D. *et al.* Sequencing of 53,831 diverse genomes from the NHLBI TOPMed Program. *Nature* **590**, 290–299 (2021).
4. Conomos, M. P., Reiner, A. P., Weir, B. S. & Thornton, T. A. Model-free Estimation of Recent Genetic Relatedness. *The American Journal of Human Genetics* **98**, 127–148 (2016).
5. Bycroft, C. *et al.* The UK Biobank resource with deep phenotyping and genomic data. *Nature* **562**, 203–209 (2018).
6. Nagai, A. *et al.* Overview of the BioBank Japan Project: Study design and profile. *J Epidemiol* **27**, S2–S8 (2017).
7. Mägi, R. & Morris, A. P. GWAMA: software for genome-wide association meta-analysis. *BMC Bioinformatics* **11**, 288 (2010).
8. Ruan, Y. *et al.* Improving polygenic prediction in ancestrally diverse populations. *Nat Genet* **54**, 573–580 (2022).
9. Euesden, J., Lewis, C. M. & O'Reilly, P. F. PRSice: Polygenic Risk Score software. *Bioinformatics* **31**, 1466–1468 (2015).
10. Huang, T. *et al.* Genetic Predisposition to Elevated C-Reactive Protein and Risk of Obstructive Sleep Apnea. *Am J Respir Crit Care Med* **209**, 329–331 (2024).
11. Chang, C. C. *et al.* Second-generation PLINK: rising to the challenge of larger and richer datasets. *Gigascience* **4**, 7 (2015).
12. Sofer, T. *et al.* A polygenic risk score for Alzheimer's disease constructed using APOE-region variants has stronger association than APOE alleles with mild cognitive impairment in Hispanic/Latino adults in the U.S. *Alzheimers Res Ther* **15**, 146 (2023).
