# Supplementary material for: Methylation risk score of C-reactive protein associates sleep health with related health outcomes
Source: Commun Biol. 2025 May 28;8:821. doi: 10.1038/s42003-025-08226-1 (PMC12119824; doi:10.1038/s42003-025-08226-1)
Supplement: Supplementary file 2 — Description of Additional Supplementary Files [file 42003_2025_8226_MOESM2_ESM.docx]

Description of Additional Supplementary Files

**File name:** Supplementary Data 1

**Description: Model 1 association results of blood-CRP, methylation (MRS-CRP) and polygenic risk score (PRS-CRP) for C-reactive protein (CRP)**
