# Supplementary material for: Methylation risk score of C-reactive protein associates sleep health with related health outcomes
Source: Commun Biol. 2025 May 28;8:821. doi: 10.1038/s42003-025-08226-1 (PMC12119824; doi:10.1038/s42003-025-08226-1)
Supplement: Supplementary file 4 — Reporting Summary [file 42003_2025_8226_MOESM4_ESM.pdf]

nature portfolio

Corresponding author(s): Ziqing Wang, Tamar Sofer

Last updated by author(s): Sep 16, 2024

Reporting Summary

Nature Portfolio wishes to improve the reproducibility of the work that we publish. This form provides structure for consistency and transparency in reporting. For further information on Nature Portfolio policies, see our Editorial Policies and the Editorial Policy Checklist.

Statistics

For all statistical analyses, confirm that the following items are present in the figure legend, table legend, main text, or Methods section.

n/a	Confirmed	
☐	☒	The exact sample size (*n*) for each experimental group/condition, given as a discrete number and unit of measurement
☐	☒	A statement on whether measurements were taken from distinct samples or whether the same sample was measured repeatedly
☐	☒	The statistical test(s) used AND whether they are one- or two-sided<br>*Only common tests should be described solely by name; describe more complex techniques in the Methods section.*
☐	☒	A description of all covariates tested
☐	☒	A description of any assumptions or corrections, such as tests of normality and adjustment for multiple comparisons
☐	☒	A full description of the statistical parameters including central tendency (e.g. means) or other basic estimates (e.g. regression coefficient) AND variation (e.g. standard deviation) or associated estimates of uncertainty (e.g. confidence intervals)
☐	☒	For null hypothesis testing, the test statistic (e.g. *F*, *t*, *r*) with confidence intervals, effect sizes, degrees of freedom and *P* value noted<br>*Give P values as exact values whenever suitable.*
☒	☐	For Bayesian analysis, information on the choice of priors and Markov chain Monte Carlo settings
☒	☐	For hierarchical and complex designs, identification of the appropriate level for tests and full reporting of outcomes
☐	☒	Estimates of effect sizes (e.g. Cohen's *d*, Pearson's *r*), indicating how they were calculated

Our web collection on statistics for biologists contains articles on many of the points above.

Software and code

Policy information about availability of computer code

Data collection	We used data that were previously collected and prepared by MESA (dbGaP repository study accession: “phs001211”) and HCHS/SOL - http://www.cscc.unc.edu/hchs/.
Data analysis	Methylation risk scores were constructed using MethParquet package (v0.1.0). Association analyses were performed using survey linear regression models (survey package v4.2.1). Penalized cubic spline models were performed using mgcv package (v1.9.1). All analysis was performed in R (v4.3.1).

For manuscripts utilizing custom algorithms or software that are central to the research but not yet described in published literature, software must be made available to editors and reviewers. We strongly encourage code deposition in a community repository (e.g. GitHub). See the Nature Portfolio guidelines for submitting code & software for further information.

Data

Policy information about availability of data

All manuscripts must include a data availability statement. This statement should provide the following information, where applicable:

- Accession codes, unique identifiers, or web links for publicly available datasets
- A description of any restrictions on data availability
- For clinical datasets or third party data, please ensure that the statement adheres to our policy

HCHS/SOL data are available through application to the data base of genotypes and phenotypes (dbGaP) accession phs000810, or via data use agreement with the

HCHS/SOL Data Coordinating Center (DCC) at the University of North Carolina at Chapel Hill, see collaborators website: https://sites.cscc.unc.edu/hchs/.
TOPMed-MESA freeze 10 WGS data are available by application to dbGaP according to the study accession: "phs001211". MESA phenotype data are available from dbGaP according to the MESA accession "phs000209".
Summary statistics from GWAS of CRP were downloaded from the PAN-UKBB project (https://pan.ukbb.broadinstitute.org/; phenocode 30710) and BioBank Japan PheWeb at https://pheweb.jp/pheno/CRP.
PRS-CRP alleles and weights will be provided at the Github repository: https://github.com/ZWangTen/CRP_markers_association.

Research involving human participants, their data, or biological material

Policy information about studies with human participants or human data. See also policy information about sex, gender (identity/presentation), and sexual orientation and race, ethnicity and racism.

Reporting on sex and gender	Sociodemographic and lifestyle variables were self-reported, including sex for MESA and HCHS/SOL. Sex was reported in the manuscript in Table 1 and adjusted for as a covariate in the analysis.
Reporting on race, ethnicity, or other socially relevant groupings	Sociodemographic and lifestyle variables were self-reported, including self-identified Hispanic background for HCHS/SOL and self-identified race groups for MESA. Hispanic background for HCHS/SOL was reported in Table 1. Race groups for MESA were reported in Table S1. Race and ethnicity groupings were not used as covariates in the association analysis.
Population characteristics	Population characteristics were described and reported in Table 1 for HCHS/SOL and Table S1 for MESA.
Recruitment	The MESA is a multi-center longitudinal cohort study comprising 6,814 men and women aged 45–84 years, self-identified from four race/ethnic groups at the time of enrollment (Exam 1; 2000-2002) with appropriate consent.<br>The HCHS/SOL is a population-based cohort study with participants of participants who were recruited from four metropolitan areas across the United States (Bronx NY, Chicago IL, Miami FL, and San Diego CA).
Ethics oversight	HCHS/SOL: This study was approved by the institutional review boards (IRBs) at each field center, where all participants gave written informed consent, and by the Non-Biomedical IRB at the University of North Carolina at Chapel Hill, to the HCHS/SOL Data Coordinating Center. All IRBs approving the study are: Non-Biomedical IRB at the University of North Carolina at Chapel Hill. Chapel Hill, NC; Einstein IRB at the Albert Einstein College of Medicine of Yeshiva University. Bronx, NY; IRB at Office for the Protection of Research Subjects (OPRS), University of Illinois at Chicago. Chicago, IL; Human Subject Research Office, University of Miami. Miami, FL; Institutional Review Board of San Diego State University. San Diego, CA.<br>MESA: All MESA participants provided written informed consent, and the study was approved by the Institutional Review Boards at The Lundquist Institute (formerly Los Angeles BioMedical Research Institute) at Harbor-UCLA Medical Center, University of Washington, Wake Forest School of Medicine, Northwestern University, University of Minnesota, Columbia University, and Johns Hopkins University.<br>This work was approved by the Beth Israel Deaconess Medical Center Committee on Clinical Investigators (protocol #2023P000538).

Note that full information on the approval of the study protocol must also be provided in the manuscript.

Field-specific reporting

Please select the one below that is the best fit for your research. If you are not sure, read the appropriate sections before making your selection.

☒ Life sciences ☐ Behavioural & social sciences ☐ Ecological, evolutionary & environmental sciences

For a reference copy of the document with all sections, see nature.com/documents/nr-reporting-summary-flat.pdf

Life sciences study design

All studies must disclose on these points even when the disclosure is negative.

Sample size	Sample size was determined based on data availability. Among the total 16,415 participants, 2,221 from the Study of Latinos-Investigation of Neurocognitive Aging (SOL-INCA) ancillary study were considered for inclusion due to available genetic data and data on methylation, sleep phenotypes and circulating C-Reactive Protein.
Data exclusions	No data were excluded.
Replication	We don't have another independent cohort data for replication analysis.
Randomization	This is not relevant to our study as we were performing association analysis.
Blinding	This is not relevant to our study as we were performing association analysis.

Reporting for specific materials, systems and methods

We require information from authors about some types of materials, experimental systems and methods used in many studies. Here, indicate whether each material, system or method listed is relevant to your study. If you are not sure if a list item applies to your research, read the appropriate section before selecting a response.

Materials & experimental systems

n/a	Involved in the study
☒	☐ Antibodies
☒	☐ Eukaryotic cell lines
☒	☐ Palaeontology and archaeology
☒	☐ Animals and other organisms
☒	☐ Clinical data
☒	☐ Dual use research of concern
☒	☐ Plants

Methods

n/a	Involved in the study
☒	☐ ChIP-seq
☒	☐ Flow cytometry
☒	☐ MRI-based neuroimaging

Plants

Seed stocks	*Report on the source of all seed stocks or other plant material used. If applicable, state the seed stock centre and catalogue number. If plant specimens were collected from the field, describe the collection location, date and sampling procedures.*
Novel plant genotypes	*Describe the methods by which all novel plant genotypes were produced. This includes those generated by transgenic approaches, gene editing, chemical/radiation-based mutagenesis and hybridization. For transgenic lines, describe the transformation method, the number of independent lines analyzed and the generation upon which experiments were performed. For gene-edited lines, describe the editor used, the endogenous sequence targeted for editing, the targeting guide RNA sequence (if applicable) and how the editor was applied.*
Authentication	*Describe any authentication procedures for each seed stock used or novel genotype generated. Describe any experiments used to assess the effect of a mutation and, where applicable, how potential secondary effects (e.g. second site T-DNA insertions, mosiacism, off-target gene editing) were examined.*
